# TPD52L2 as a potential prognostic and immunotherapy biomarker in clear cell renal cell carcinoma

**DOI:** 10.3389/fonc.2023.1210910

**Published:** 2023-11-23

**Authors:** Hongbo Wang, Zhendong Liu, Yuelin Du, Xingbo Cheng, Shanjun Gao, Yanzheng Gao, Panfeng Shang

**Affiliations:** ^1^ Department of Urology Surgery, Lanzhou University Second Hospital, Lanzhou, China; ^2^ Department of Surgery of Spine and Spinal Cord, Henan Provincial People’s Hospital, People’s Hospital of Zhengzhou University, Zhengzhou, China; ^3^ Department of Microbiome Laboratory, Henan Provincial People’s Hospital, People’s Hospital of Zhengzhou University, Zhengzhou University, Zhengzhou, China

**Keywords:** clear cell renal cell carcinoma, TPD52 Like 2, prognosis, tumor microenvironment, malignant phenotype

## Abstract

**Background:**

Tumor Protein D52-Like 2 (TPD52L2) is a tumor-associated protein that participates in B-cell differentiation. However, the role of TPD52L2 in the pathological process of clear cell renal cell carcinoma (ccRCC) is unclear.

**Methods:**

Multiple omics data of ccRCC samples were obtained from public databases, and 5 pairs of ccRCC tissue samples were collected from the operating room. Wilcox, chi-square test, Kaplan-Meier method, receiver operating characteristic curve, regression analysis, meta-analysis, and correlation analysis were used to clarify the relationship of TPD52L2 with clinical features, prognosis, and immune microenvironment. Functional enrichment analysis was performed to reveal the potential pathways in which TPD52L2 participates in the progression of ccRCC. The siRNA technique was used to knockdown in the expression level of TPD52L2 in 786-O cells to verify its effect on ccRCC progression.

**Results:**

First, TPD52L2 was found to be upregulated in ccRCC at both mRNA and protein levels. Second, TPD52L2 was significantly associated with poor prognosis and served as an independent prognostic factor. Moreover, TPD52L2 expression was regulated by DNA methylation, and some methylation sites were associated with ccRCC prognosis. Third, TPD52L2 overexpression may participate in the pathological process through various signaling pathways such as cytokine-cytokine receptor interactions, PI3K-Akt, IL-17, Wnt, Hippo signaling pathway, and ECM-receptor interactions. Interestingly, TPD52L2 expression level was also closely related to the abundance of various immune cells, immune checkpoint expression, and TMB. Finally, *in vitro* experiments confirmed that knocking down TPD52L2 can inhibit the proliferation, migration, and invasion abilities of ccRCC cells.

**Conclusion:**

This study for the first time revealed the upregulation of TPD52L2 expression in ccRCC, which is closely associated with poor prognosis of patients and is a potentially valuable therapeutic and efficacy assessment target for immunotherapy.

## 1 Introduction

Clear cell renal cell carcinoma (ccRCC) is the most common histological type of renal cell carcinoma (1). Currently, the mainstay of treatment for early-stage or localized ccRCC is radical nephrectomy, which has a better prognosis (2). However, because ccRCC often grows insidiously, 17% patients have already developed metastatic tumors when diagnosed (3). Although surgical treatment, targeted therapy, immunotherapy, and other comprehensive treatment modalities have been adopted for advanced ccRCC, the median survival of patients is only 19 months (3). What’s more, there are many problems such as postoperative recurrence, targeted drug resistance, and uncertain response to immunotherapy (4). The main reason for this dilemma is that the mechanisms underlying ccRCC initiation and progression have not been fully elucidated, and there is a lack of effective biomarkers for prognosis and individualized diagnosis and treatment.

It is well known aberrant oncogene or oncogene expression not only affects the prognosis of ccRCC patients but is also closely associated with alterations in the tumor immune microenvironment (TME). For example, the abnormally elevated expression of CCL5 in ccRCC tissues may promote the malignant progression of ccRCC by regulating the polarization state of tumor-associated macrophages and participating in the remodeling of the TME, leading to a poor prognosis; Concurrently, the expression level of CCL5 is positively correlated with the infiltrating abundance of CD8+ T cells and the expression of immune checkpoints, which are potential diagnostic and therapeutic biomarkers (5). Nevertheless, existing biomarkers are still insufficient to adequately address the early diagnostic and therapeutic needs of ccRCC. Consequently, there is an urgent need to identify new biomarkers to better serve clinical practice.

TPD52L2, a member of the tumor protein D52 family, is abnormally high expressed in glioma, gastric cancer, breast cancer and other tumor tissues, and plays an important role in regulating the proliferation, invasion, migration, and other malignant biological behaviors of tumor cells, accelerating the malignant pathological process of tumors (6–8). In the pathological process of malignant tumors, tumor cells and immune cells, stromal cells through mutual secretion of a variety of cytokines, achieve cellular communication to form a suppressive immune molecular network, and ultimately achieve the immune escape of tumor cells (9). And the important function of TPD52 protein family is to participate in the regulation of Ca2^+^ dependent vesicular transport process and the differentiation of B cells (10). Whether this implies that TPD52L2 plays a potential role in shaping the TME remains to be determined. Some scholars have focused on this, such as Zhong et al. confirmed that TPD52L2 is an oncogene with abnormally higher expression in lung adenocarcinoma and is involved in the formation of a suppressive immune microenvironment (6). ccRCC is a highly immunogenic and highly immunoreactive tumor. Does TPD52L2 have an impact on the occurrence and development of ccRCC? Unfortunately, this has not been systematically studied, which piqued our interest as a ccRCC researcher.

Therefore, in the present study, we sought to unravel the role played by TPD52L2 in the pathological progression of ccRCC through a comprehensive analysis of multiple data sources. To our knowledge, the present study is the first to explore the effect of TPD52L2 on the prognosis of ccRCC patients, and the association of TPD52L2 with immune infiltration and immunotherapy. In conclusion, this study contributes to a deeper understanding of the function of TPD52L2 and provides new directions for the diagnosis, prognostic assessment, and immune combination therapy of ccRCC.

## 2 Methods

### 2.1 Data access

The current study was conducted in multiple dimensions, from proteome to transcriptome, to obtain a comprehensive and reliable result. First, the Tumor Immune Estimation Resource (TIMER, https://cistrome.shinyapps.io/timer/) database was used to explore the expression levels of TPD52L2 in a variety of tumors (11). Second, to explore the impact of TPD52L2 on the prognosis of ccRCC patients and the role it plays in the pathological course of the disease, this study obtained transcriptomic and methylation data from The Cancer Genome Atlas (TGCA), Gene Expression Omnibus (GEO), International Cancer Genome Consortium (ICGC), and Array Express public data platforms for patients. Transcriptome data for 537 ccRCC patients and methylation data for 317 patients were obtained from TCGA (https://portal.gdc.cancer.gov/) (12), and specific clinical information is presented in **Tables S1, S2**. Of these patients,72 samples matched normal tissues. Then, 4 ccRCC transcriptome datasets were obtained from GEO (https://www.ncbi.nlm.nih.gov/geo/) (13): GSE36895 (containing 23 normal and 29 tumor samples), GSE46699 (63 normal and 67 tumor samples), GSE53757 (72 normal and 72 tumor samples), and GSE66272 (27 normal and 27 tumor samples), GSE53757 (72 normal and 72 tumor samples), GSE66272 (27 normal and 27 tumor samples), GSE22541(48 tumor samples), and GSE40912(16 tumor samples). And another sequencing data of 91 ccRCC patients were obtained through the ICGC database (https://dcc.icgc.org/). E-MTAB-1980 microarray data is obtained from the Array Express database(https://www.ebi.ac.uk/arrayexpress/), which contains the RNA seq and clinical follow-up information of 101 ccRCC patients (14). Third, the Clinical proteomic tumor analysis consortium (CPTAC, https://proteomic.datacommons.cancer.gov/pdc/) database was used to obtain 110 ccRCC tissues and 84 normal tissue samples were used to clarify the protein expression level of TPD52L2 in ccRCC. In addition, immunohistochemical staining of TPD52L2 protein in ccRCC tissues was also obtained through Human Protein Atlas (HPA, https://www.proteinatlas.org/).

In addition to the above public databases, the current study collected 5 pairs of ccRCC and paraneoplastic tissues from the operating room of the Second Hospital of Lanzhou University for proteomic sequencing.

### 2.2 Cell culture and transfection

Renal clear cell carcinoma cell lines 786-O, and normal renal embryonic cells 293-T were obtained from the Institute of Urology, Second Hospital of Lanzhou University. The culture conditions were as follows:786-O: 10% fetal bovine serum (FBS, Gibco, USA) in 1640 (Procell, China) culture. 293-T: 10% FBS in DMEM (Procell, China) culture. The cells were incubated in an incubator at 37 °C with 5% CO2 and used for subsequent experiments when cell growth was approximately 90%. The small interfering RNA (siRNA) was obtained from gemma (Genepharma,China), and the specific sequences are shown in **Table S3**. The cell transfection protocol was performed according to the instructions of Lipofectamine 3000, and the cell transfection efficiency was detected by RT-qPCR after 36h transfection.

### 2.3 Real-timeime quantitative PCR

The total RNA was extracted by Total RNA Kit I (Omega, USA). The concentration and purity of the RNA were subsequently measured using NanoDrop (Thermo, USA). The cDNA was obtained by reverse transcription using NovoScript Plus All-in-one 1st Strand cDNA Synthesis SuperMix kit (Novoprotein, China) according to the instructions. Ultimately, RT-qPCR was used to detect the expression level of TPD52L2 by Novostart SYBR qPCR SuperMix Plus Kit (Novoprotein, China). Primer sequences required for the experiment are as follows: GAPDH-Forward: CAAGGTCATCCATGACAACTTTG and GAPDH-Reverse: TCCACCACCCTGTTGCTGTAG; TPD52L2-Forward: CTCCATGACGGATGTTCCTGT and TPD52L2-Reverse: GCCTCTGTCAGACCCTCAAC. The result calculation method adopts 2^−ΔΔCT^.

### 2.4 Meta-analysis

To further confirm the effect of TPD52L2 on the prognosis of ccRCC patients, we included multiple groups of patient data for meta-analysis. And five datasets containing survival information from different databases were included in the study (TCGA: 530 cases; E-MATB-1980: 106 cases; GSE40912: 16 cases; ICGC: 91 cases; GSE22541:48 cases). The pooled hazard ratio (HR) and 95% CI were calculated to evaluate the association of TPD52L2 expression with ccRCC patient prognosis. Heterogeneity between the different data sets was assessed by the Q test (I^2^ statistic). If there is no obvious heterogeneity(I^2^<50%), select the fixed effect model. On the contrary, the random effect model will be applied.

### 2.5 Enrichment analysis of differentially expressed genes in ccRCC

First, DEGs in the TCGA ccRCC dataset were obtained from patients in the high and low TPD52L2 groups by the limma package of R software, with |LogFC|>1, false discovery rate (FDR) <0.05. After that, GO and KEGG enrichment analysis of DEGs was performed using by cluster profile package. P-value < 0.05 was considered to have in significant enrichment difference.

### 2.6 Gene set enrichment analysis

To further explore the potential biological processes and pathways involved in the pathological progression of ccRCC by TPD52L2, gene set enrichment analysis (GSEA) was also performed in this study. First, the TCGA-ccRCC dataset was divided into high and low-expression groups based on the median expression of TPD52L2. After that, enrichment analysis was performed using GSEA software (version: 4.2.1). The related settings are as follows: Gene sets database is set to c2.cp.kegg. v7.5.1; The number of permutations is set to 1000. P-value < 0.05 and |NES|>1 was considered statistically significant.

### 2.7 Immune correlation analysis of TPD52L2 in ccRCC

The ESTIMATE algorithm is a popular tool for tumor immunology research, which not only calculates the percentage of immune and stromal cell infiltration in tumor samples based on gene expression data but also calculates tumor purity (15). To clarify the relationship between TPD52L2 expression levels and immune cell infiltration, we analyzed the degree of immune cell infiltration between the TCGA-ccRCC samples in the high and low TPD52L2 expression groups using the ESTIMATE package in R language and compared the differences between the two groups using the Wilcoxon test. CIBERSORT and ssGSEA are widely used methods to quantify the infiltration status of different immune cells (16, 17). The matrix of high and low-expression TPD52L2 expression profiles was analyzed using these two methods, which in turn led to the infiltration pattern of different immune cells and immune activities in the two groups of samples, using the Wilcoxon test for differences between groups.

### 2.8 Immunotherapy effect analysis

Currently, the expression level of immune checkpoints represented by PD-L1 and tumor mutation burden (TMB) are important reference indicators for assessing the effect of immunotherapy in tumor patients (18, 19). In this study, we attempted to indirectly investigate the role of TPD52L2 in the assessment of immunotherapy efficacy in ccRCC patients by comparing the differences in immune checkpoint expression and TMB between two groups of patients with high and low TPD52L2 expression. The cancer immunome Atlas (TCIA, https://tcia.at) is a tumor immune profiling tool developed based on the TCGA database (20). The immunophenoscore (IPS), developed by this database, is a good predictor of anti-CTLA4 and anti-PD-1 antibody responses. To further assess the effect of TPD52L2 expression levels on the efficacy of immunotherapy, we obtained IPS from ccRCC patients from TCIA and compared the differences between the two groups.

### 2.9 CCK8

After transfecting the cells, they were seeded into 96-well plates at a density of 4 × 10^3 cells per well. Cell proliferation was assessed at four time points, namely 0h, 24h, 48h, and 72h. Specifically, 10μl of CCK-8 reagent (Beyotime Biotechnology) was added to each well, followed by incubation at 37°C for 1 hour. The optical density was then measured at 450 nm using an enzyme-linked immunosorbent assay reader.

### 2.10 Immunofluorescence

Immunofluorescence staining was performed to detect cell proliferation and apoptosis. Cells were fixed with 4% paraformaldehyde, permeabilized with 0.1% Triton X-100, and blocked with 5% BSA. The cells were then incubated with primary antibody against Ki-67(1:200, Abcam) followed by a secondary antibody conjugated with red fluorescent dye. DAPI staining was used to visualize the nuclei. Fluorescence images were captured using a fluorescence microscope. Apoptosis detection was done with Hoechst 33258 Apoptosis Staining Kit (Beyotime Biotechnology). After fixing the cells with 4% paraformaldehyde, the morphological changes of the nuclei were observed by fluorescence microscopy after adding the Hoechst 33258 staining solution. The experimental results were analyzed using Image J.

### 2.11 Wound healing assay

The wound healing assay was used to detect changes in the migratory ability of cells. First, transfected cells were seeded into a 6-well plate and allowed to proliferate to 90%-100% confluence. A 200 μl pipette tip was used to create a scratch of uniform width in each well. The original culture medium was removed, and the wells were washed three times with PBS before adding serum-free basal medium. Pictures were taken and recorded at 0 and 12 hours.

### 2.12 Transwell migration assay

An 8 μm Transwell chamber was used. Complete culture medium containing 10% FBS was added to the lower chamber, and no medium was added to the upper chamber. Transfected cells were digested and resuspended in serum-free basal medium at a density of 3x10^5 cells/ml. A volume of 100 μl of the cell suspension was added to the upper chamber of each Transwell. The Transwells were then incubated at 37°C for 12-24 hours. The original medium was removed, and the wells were washed three times with PBS before fixing with 4% paraformaldehyde for 30 minutes. After staining with 0.5% crystal violet for 30 minutes, pictures were taken under a microscope and the number of migrated cells was calculated.

### 2.13 Transwell invasion assay

An 8 μm Transwell chamber was used. First, 50 μl of a mixture of matrix gel and serum-free basal medium at a ratio of 1:8 was added to the upper chamber of each Transwell and incubated for 3 hours to allow the matrix gel to solidify. Complete culture medium containing 10% FBS was then added to the lower chamber. Transfected cells were digested and resuspended in serum-free basal medium at a density of 3x10^5 cells/ml. A volume of 100μl of the cell suspension was added to the upper chamber of each Transwell. The Transwells were then incubated at 37°C for 36 hours. The original medium was removed, and the wells were washed three times with PBS before fixing with 4% paraformaldehyde for 30 minutes. After staining with 0.5% crystal violet for 30 minutes, pictures were taken under a microscope and the number of invaded cells was calculated.

### 2.14 Statistical analysis

All statistical analyses were performed using R software (version: 4.1.1). The results of RT-qPCR were compared using an unpaired *t-*test. Differences in the results of proteomic sequencing of TPD52L2 were compared using paired *t*-tests. Survival curves were drawn using the Kaplan-Meier method, and differences were evaluated using the log-rank-test method. The receiver operating characteristic (ROC) curve was used to evaluate the diagnostic value of TPD52L2 for patient prognosis. Univariate and multivariate Cox survival regression analysis was used to determine whether TPD52L2 could be used as an independent prognostic indicator. The Spearman correlation coefficient is used to assess the correlation between two variables. Statistical significance was considered when *P* < 0.05.

## 3 Results

### 3.1 TPD52L2 is aberrantly highly expressed in multiple cancers and closely associated with tumor mutational burden

**Figure 1:**
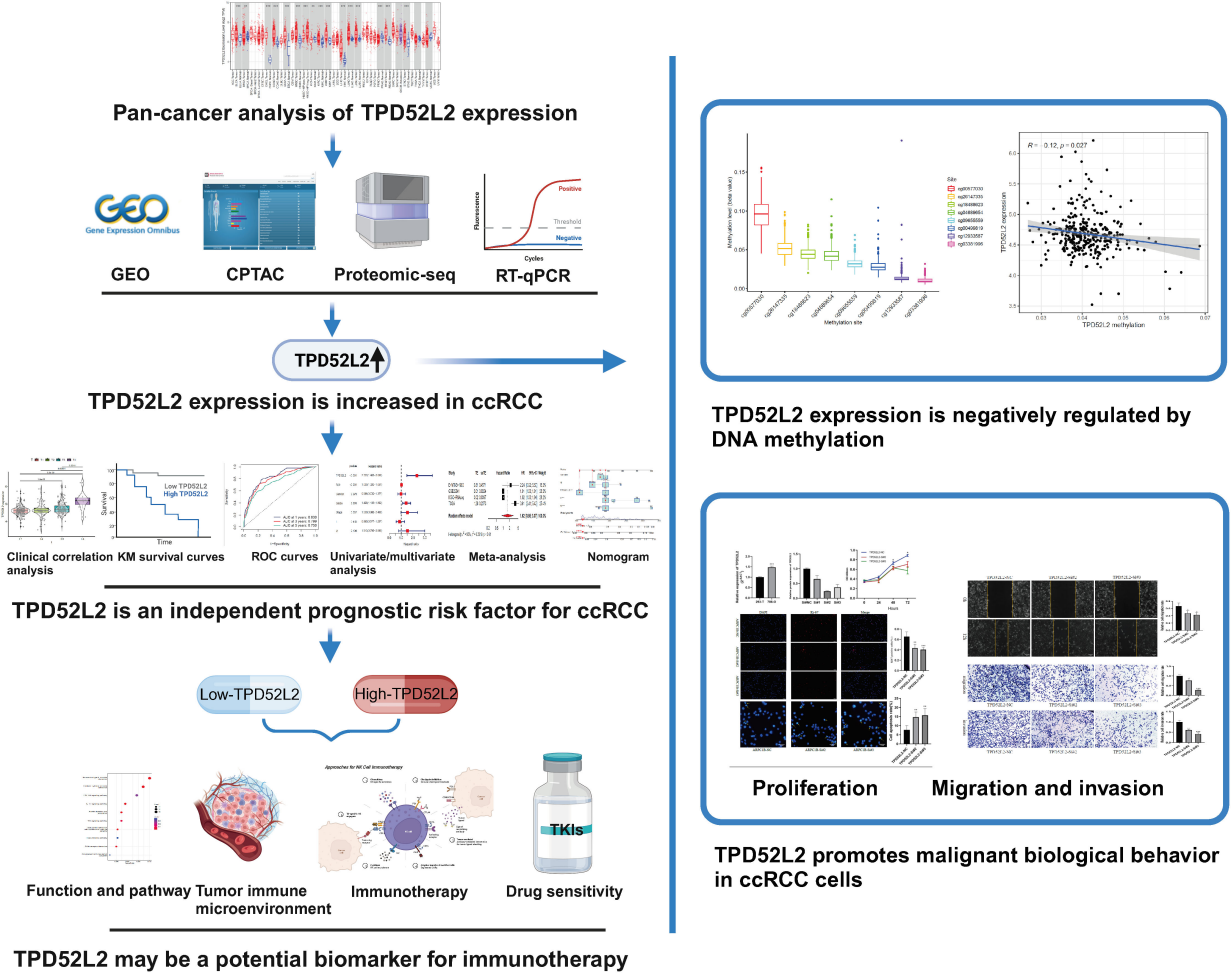
Flow chart of the study. (Created with BioRender.com). **P<0.01; ***P<0.001.

**Figure 2:**
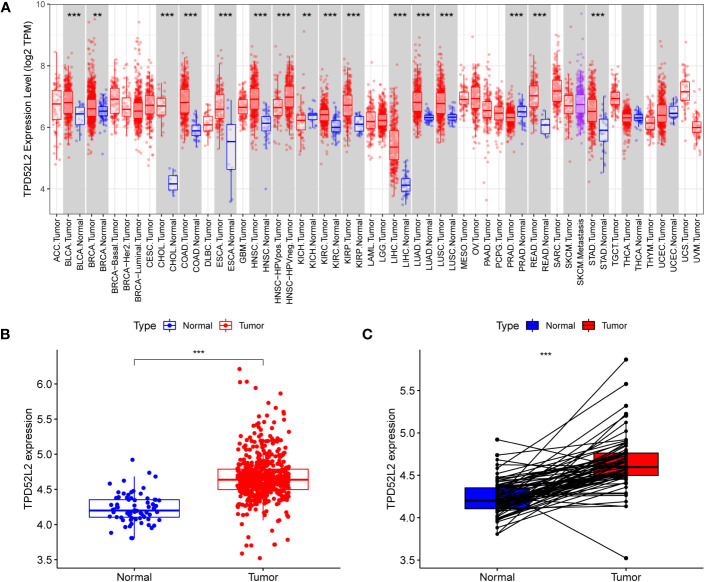
TPD52L2 expression levels in multiple tumors including renal cancer. **(A)** TPD52L2 is expressed at increased levels in a variety of tumors; **(B, C)** Expression levels of TPD52L2 in ccRCC tumors and paired tissues.

The flow diagram of our study is presented in **Figure 1**. Disrupted gene expression is one of the major hallmarks of cancer and has a major impact on tumorigenesis, growth, and progression. To initially clarify whether TPD52L2 might be an oncogene, its expression pattern in different tumors was first confirmed in this study. The results shown in **Figure 2A**, TPD52L2 was highly expressed in Kidney renal clear cell carcinoma, Kidney renal papillary cell carcinoma, Liver hepatocellular carcinoma, Bladder Urothelial Carcinoma, Breast invasive carcinoma, Cholangio carcinoma, Colon adenocarcinoma, Esophageal carcinoma, Head and Neck squamous cell carcinoma, Lung adenocarcinoma, Lung squamous cell carcinoma, Rectum adenocarcinoma, and Stomach adenocarcinoma; TPD52L2 was low expressed in Kidney Chromophobe and Prostate adenocarcinoma. Its aberrant expression in ccRCC aroused our research interest. Therefore, the expression level of TPD52L2 in ccRCC paired as well as unpaired tissues were further explored, and the results are shown in **Figures 2B, C**. In addition, this study also attempted to explore the relationship between TPD52L2 and TMB, an important marker of tumor immunotherapy response. The results suggest that TPD52L2 is positively correlated with TMB in various tumors including ccRCC (**Figure S1**). Taken together, the above results indicate that TPD52L2 expression is elevated in ccRCC and may have a potential predictive role for tumor immunotherapy.

### 3.2 Multiple perspectives confirm TPD52L2 expression is abnormally increased in ccRCC

**Figure 3:**
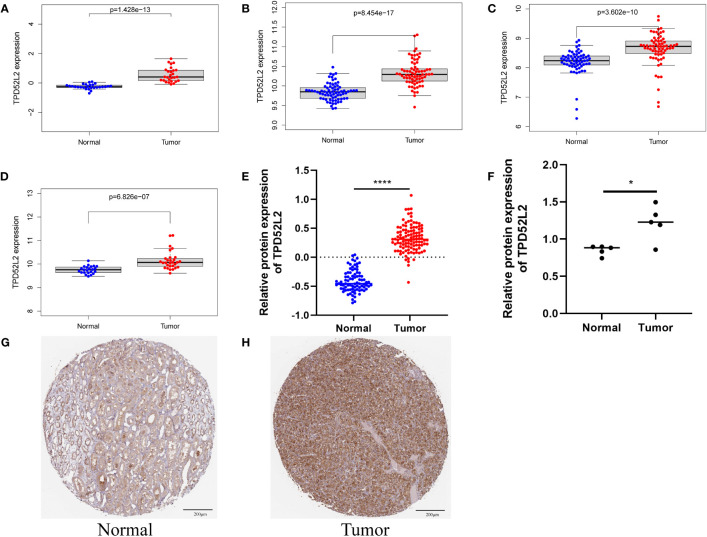
The mRNA and protein expression patterns of TPD52L2 in ccRCC tissues and cells. **(A–D)** TPD52L2 is up regulated in ccRCC tissues based on GSE36895, GSE46699, GSE57357 and GSE66272, respectively; **(E)** Differential protein expression of TPD52L2 in ccRCC tissues based on CPTAC; **(F)** Differences of TPD52L2 in 5 pairs of ccRCC and adjacent tissues proteomic sequencing results; **(G, H)** Protein expression of TPD52L2 based on immunohistochemical results in HPA. *P<0.05, ****P<0.0001.

To further determine whether TPD52L2 plays a potentially important role in the paleopathology of ccRCC, we conducted an in-depth and comprehensive study. In this study, we first confirmed the expression level of TPD52L2 in ccRCC cells and tissues by mRNA and protein levels. First, at the mRNA level, TPD52L2 expression was demonstrated to be elevated in ccRCC tissues through GSE66272, GSE53757, GSE46699, and GSE36895 microarray data of a total of 195 RCC tissues, as shown in **Figures 3A–D**.

In addition, the protein level of TPD52L2 was also verified. First, the protein level of TPD52L2 was abnormally high expressed in 110 ccRCC proteomic sequencing data, as shown in **Figure 3E**.

Further, the proteomic sequencing data of 5 pairs of ccRCC tissue samples from our study were also analyzed, and the results showed the protein expression level of TPD52L2 in tumor tissues was still abnormally increased (**Figure 3F**). Immunohistochemical results showed that the expression of TPD52L2 in ccRCC samples was higher than that in normal samples (**Figures 3G, H**). In short, the above studies show that the expression of TPD52L2 is abnormally increased in ccRCC tissues and cells, which may play an important role in the pathological process of ccRCC.

### 3.3 TPD52L2 expression is closely associated with multiple malignant clinical features of ccRCC

**Figure 4:**
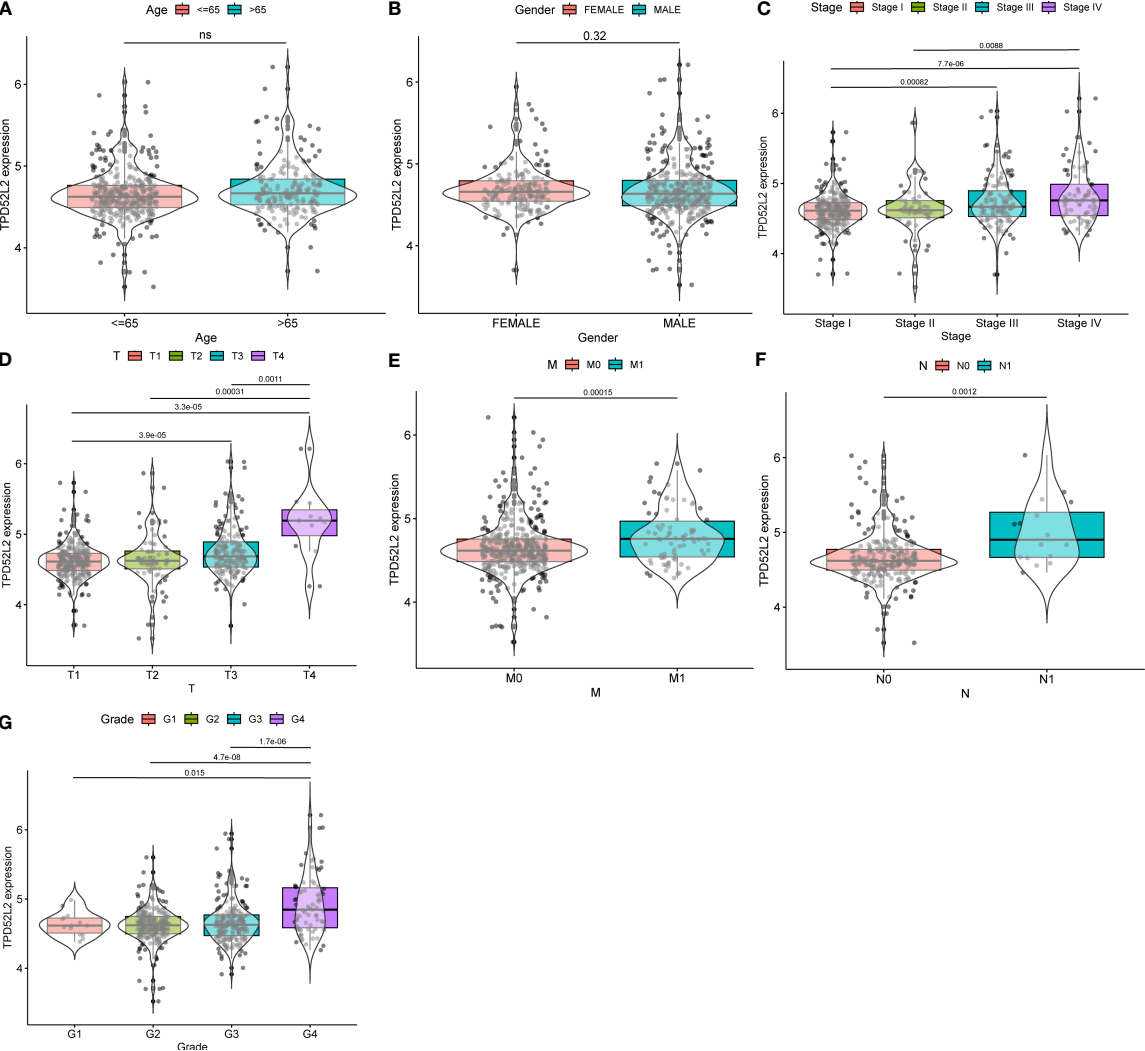
The relationship between TPD52L2 expression and clinical characteristics of ccRCC based on TCGA. **(A)** Age, **(B)** Gender, **(C)** Stage, **(D)** T stage, **(E)** M stage, **(F)** N stage, **(G)** Grade. ns, no significance.

Previous studies have shown that the process of malignant tumor initiation and progression is often accompanied by the disturbance of gene expression patterns, and the expression levels of oncogenic genes are often closely related to malignant pathological features. To explore whether TPD52L2 is a potential oncogene in ccRCC, the expression level of TPD52L2 was analyzed in relation to the clinical characteristics of ccRCC patients based on sequencing data from TCGA. The results showed that there was no significant difference in the expression of TPD52L2 between different ages and sexes, as shown in **Figures 4A, B.** The expression level of TPD52L2 increases with the increase of tumor TMN stage and grade, as shown in **Figures 4C–G**. The expression level of TPD52L2 is positively correlated with the malignant clinical features, which also indicates that may play the role of a pathogenic gene in the pathological process of ccRCC and may also have a specific impact on patient diagnosis.

### 3.4 TPD52L2 is an independent prognostic factor for patients with ccRCC

**Figure 5:**
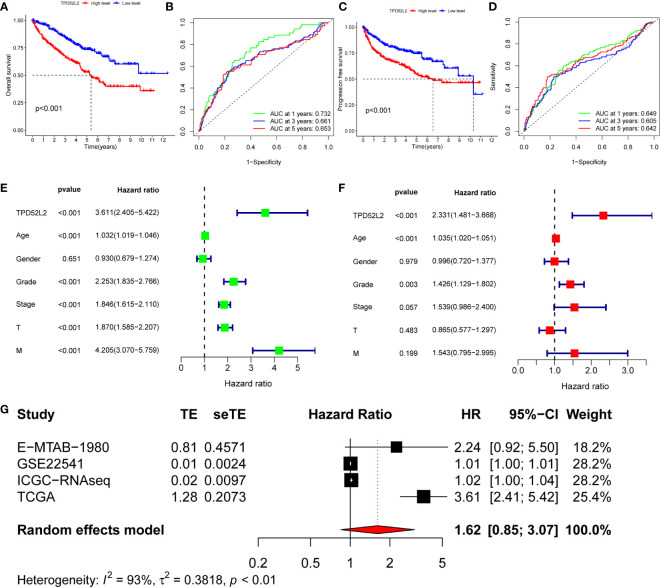
The value of TPD52L2 on the prognosis of ccRCC patients. **(A)** Relationship between TPD52L2 expression and overall survival (OS) of ccRCC; **(B)** Diagnostic value of TPD52L2 on 1-year, 3-year, and 5-year OS in ccRCC patients; **(C)** Relationship between TPD52L2 expression and progression free survival (PFS) in ccRCC patients; **(D)** Diagnostic value of TPD52L2 on 1-year, 3-year, and 5-year PFS in ccRCC patients; **(E)** Univariate analysis on clinical characteristics of ccRCC based on TCGA; **(F)** Multivariate analysis on clinical characteristics of ccRCC based on TCGA; **(G)** The meta-analysis of TPD52L2 prognostic value based on TCGA, GSE22541,ICGC and E−MTAB−1980.

The above research results show that TPD52L2 is abnormally high expressed in ccRCC tissues and cells and is positively correlated with the malignant clinical characteristics of patients, which means that TPD52L2 is a potential carcinogen gene of ccRCC. To define the impact of TPD52L2 on the prognosis of ccRCC patients, we conducted comprehensive studies including survival analysis, ROC analysis, univariate analysis, multivariate analysis, and meta-analysis. First, the results of survival analysis showed that patients with high expression of TPD52L2 had shorter overall survival(OS) and progression-free progression(PFS), as shown in **Figures 5A, C**. The ROC curve of the OS shows that the AUC of TPD52L2 is 0.732, 0.661, and 0.653 at 1, 3, and 5 years, respectively (**Figure 5B**). However, the ROC curve of PFS shows that the AUC of 1, 3, and 5 years is 0.649, 0.605, and 0.642, respectively (**Figure 5D**). Given this, to further clarify whether TPD52L2 is prognostic factor, univariate and multivariate analyses were performed. Univariate analysis revealed that TPD52L2, age, tumor grade, tumor stage, T, and M were prognostic risk factors for ccRCC patients (**Figure 5E**). The results of multivariate analysis showed that TPD52L2, age, tumor grade were independent prognostic risk factors for ccRCC patients (**Figure 5F**).

In addition, to further confirm that TPD52L2 is an oncogene, this study included five data sets of E-MTAB-1980, GSE22541, GSE40912, ICGC-ccRCC, and TCGA-ccRCC for meta-analysis of 791 ccRCC. The pooled HR along with 95% CI for the relation between high TPD52L2 expression and OS in five datasets of ccRCC patients was 1.45 (0.87-2.52), as shown in **Figure 5G**. Based on the above results, we can confidently determine that TPD52L2 is a reliable prognostic biomarker for ccRCC patients and has certain diagnostic value. Collectively, we are convinced that TPD52L2 plays an oncogene role in the pathological process of ccRCC. However, what mechanism causes the increased expression of TPD52L2 remains unclear.

### 3.5 The utilization of a TPD52L2-based nomogram demonstrates a dependable impact on the prognostic prediction of ccRCC

**Figure 6:**
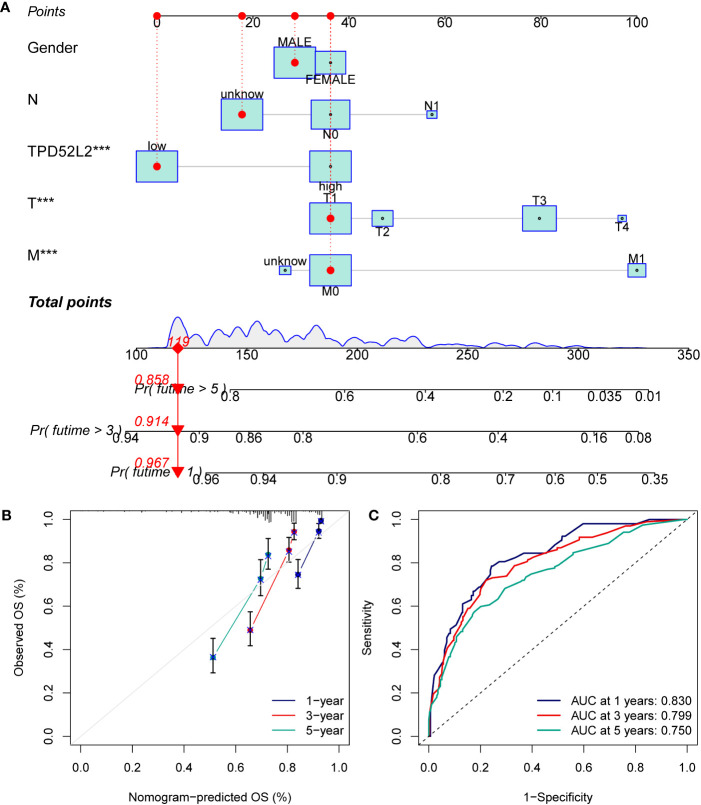
Constructing and validating nomograms. **(A)** Nomogram for predicting the 1,3 and 5-year OS of ccRCC patients; **(B)** calibration of the nomogram at 1,3 and 5-year survival; **(C)** ROC curves to predict the sensitivity and specificity of 1-, 3-, and 5-year OS according to the nomogram. ***P<0.001.

The aforementioned studies have demonstrated TPD52L2 serves as an independent prognostic risk factor for ccRCC. However, it is important to note that the prognosis of ccRCC patients is influenced by various factors. Consequently, this study incorporated both the clinical information of ccRCC patients and TPD52L2 expression to develop a nomogram, which aims to enhance prognostic assessment (**Figure 6A**). To assess the efficacy of the nomogram, a calibration curve was constructed. The findings revealed a strong concordance between the predicted 1, 3, and 5-year prognosis rates generated by the nomogram and the actual survival rate of ccRCC patients, as depicted in **Figure 6B**. Additionally, the results of the ROC curve showed for the 1-year, 3-year, and 5-year prognosis predicted by the nomogram, the AUC values were 0.83, 0.799, and 0.750, respectively (**Figure 6C**). This indicated that the prognostic nomogram constructed in this study based on TPD52L2 expression had a better predictive effect.

### 3.6 DNA methylation regulates TPD52L2 expression and affects ccRCC patient prognosis

**Figure 7:**
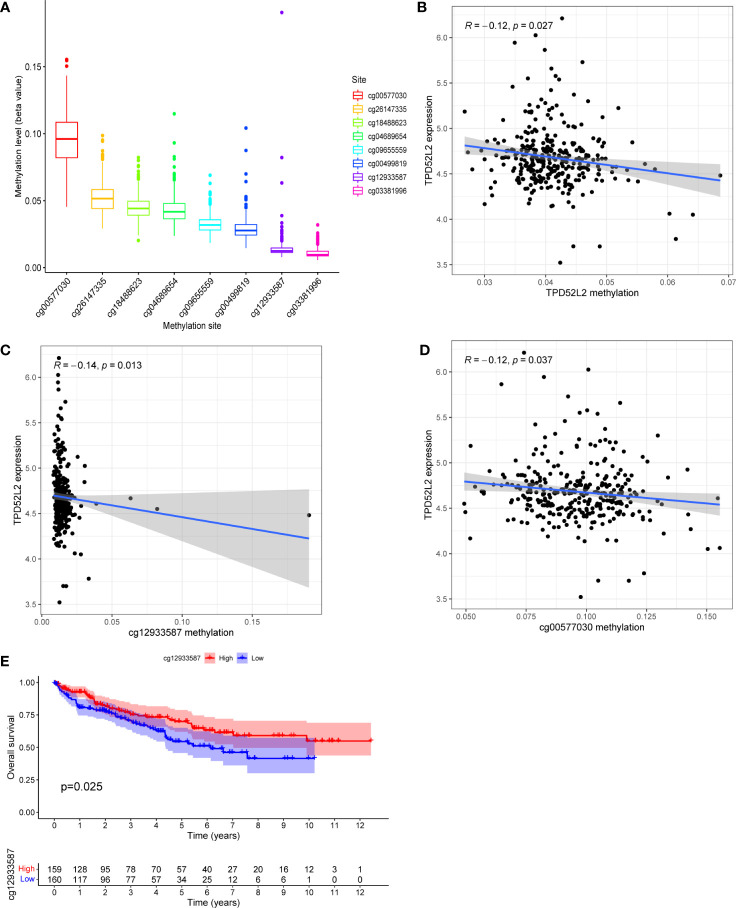
TPD52L2 expression levels are regulated by DNA methylation. **(A)** The DNA methylation levels of 8 CpG sites of gene TPD52L2; **(B)** The expression level of TPD52L2 is negatively regulated by its methylation level; **(C, D)** The expression level of TPD52L2 is negatively regulated by 2 methylation sites; **(E)** Patients with high methylation levels of cg12933587 have a better prognosis.

DNA methylation, one of the common ways of gene epigenetic modification, plays a key role in the process of tumorigenesis and development (21). It can regulate gene expression by recruiting proteins or transcription factors that repress gene expression (22). To attempt to clarify what mechanistic regulation TPD52L2 is subjected to resulting in elevated mRNA expression, the methylation information of TPD52L2 in ccRCC tissues was obtained from TCGA for further investigation in this study. After analysis, 8 DNA methylation sites regulating TPD52L2 were found (**Figure 7A**). Pearson correlation analysis showed that the mRNA expression level of TPD52L2 was negatively correlated with the methylation of TPD52L2 (**Figure 7B**). Further analysis of the 8 methylation sites found that the low methylation levels of cg12933587 and cg00577030 were closely associated with the increased expression levels of TPD52L2 (**Figures 7C, D**). Meanwhile, KM survival analysis revealed that the hypomethylation level of cg12933587 was associated with poor prognosis of patients (**Figure 7E**). The above studies suggest that the expression level of TPD52L2 in ccRCC is regulated by TPD52L2 methylation and that the methylation level of cg12933587 correlates with patient prognosis and may be a potential prognostic indicator.

### 3.7 Enrichment analysis of TPD52L2 in ccRCC

**Figure 8:**
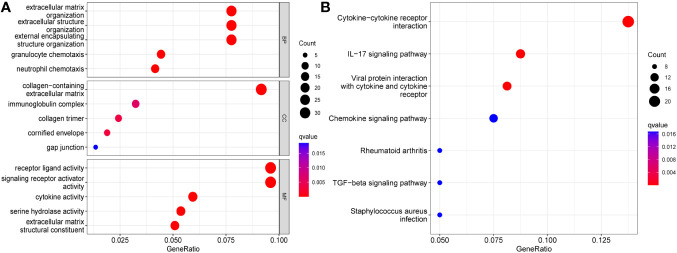
Functional enrichment analysis results of TPD52L2. **(A)** GO analysis; **(B)** KEGG analysis.

Changes in the expression of individual genes are often involved in the pathological process of the disease by triggering changes in regulatory axes or signaling pathways. To clarify the role played by TPD52L2 in the pathological process of ccRCC, a functional enrichment analysis was performed in this study. First, ccRCC samples were divided into high and low expression groups according to TPD52L2 expression. After differential analysis of the samples in the two groups, a total of 534 differentially expressed genes (DEGs) were obtained, as shown in **Data Sheet 1**. Subsequently, GO and KEGG analyses were performed on DEGs, and the results are shown in **Figures 8A, B**. GO functional analysis showed that the main biological processes enriched in DEGs were extracellular matrix and immune activity; the main cellular components included collagen and immune proteins; the main molecular functions included cell receptor activity and cytokine activity, etc. The results of KEGG pathway analysis showed that the main signaling pathways enriched by DEGs include Cytokine-cytokine receptor interactions, PI3K-Akt signaling pathway, IL-17 signaling pathway, Wnt signaling pathway, Hippo signaling pathway and ECM-receptor interactions. These pathways are involved in the invasion of tumor cells and immune regulation, indicating that the abnormally high expression of TPD52L2 plays an important role in the tumor progression and TME.

### 3.8 Relationship between TPD52L2 and immune signature

The above results suggest that TPD52L2 may be involved in regulating the TME of ccRCC.

**Figure 9:**
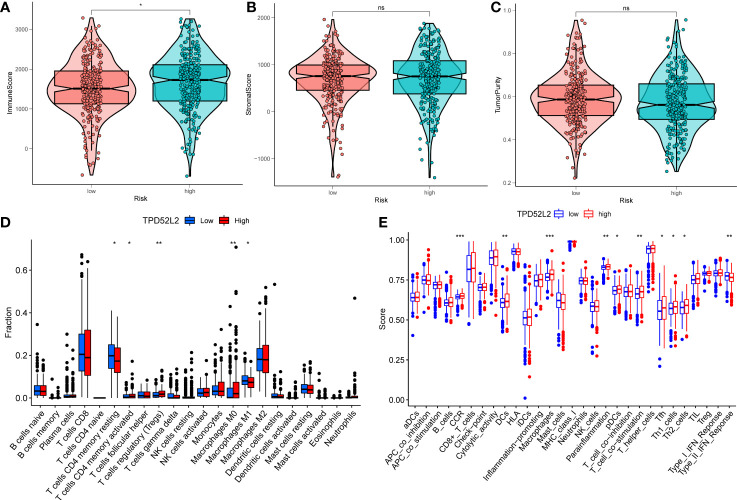
The relationship between the expression of TPD52L2 and tumor immune microenvironment and immune cell infiltration. **(A–C)** Association of TPD52L2 expression with immune cells, stromal cells, and tumor purity; **(D)** the CIBERSORT algorithm quantified 22 immune cells between the high-TPD52L2 and low-TPD52L2 groups; **(E)** analysis of differences in immune cells and immune pathways between high-TPD52L2 and low-TPD52L2 groups using ssGSEA. *P<0.05, **P<0.01, ***P<0.001. ns, no significance.

To explore this issue, this study clarified the relationship between the expression level of TPD52L2 and the level of immune cell infiltration in the immune microenvironment by ESTIMATE algorithm. The results showed that the immune score was higher in the TPD52L2 high expression group, while the tumor purity was lower (**Figure 9A**). Meanwhile, correlation analysis showed that the expression of TPD52L2 was positively correlated with immune score (**Figure S2A**). and there was no association with tumor purity with stromal cell content, as shown in **Figures 9B, C**. To further explore the effect of TPD52L2 expression and immune activity in TME, we analyzed the infiltration abundance of 22 immune cell species in ccRCC-TME using the CIBERSORT algorithm. The results showed that cells CD4 memory activated, T cells regulatory (Tregs), and Macrophages M0 infiltrated in higher abundance in the TPD52L2 high expression group, while cells CD4 memory resting and Macrophages M1 infiltrated in higher abundance in the low expression group, as shown in **Figure 9D**. Meanwhile, correlation analysis showed that TPD52L2 expression was positively correlated with the abundance of Tregs and T cells CD4 memory activated infiltration, and negatively correlated with the degree of T cells CD4 memory resting and Macrophages M1 infiltration, as shown in **Figures S2D–G**. In addition, the results of ssGSEA analysis also showed a higher abundance of macrophage infiltration in the TPD52L2 high expression group (**Figure 9E**). According to the above results, it can be suggested that TPD52L2 may be involved in the pathological process of the disease and affect patient prognosis by participating in the regulation of the TME of ccRCC.

### 3.9 The relationship between TPD52L2 expression and immunotherapy

**Figure 10:**
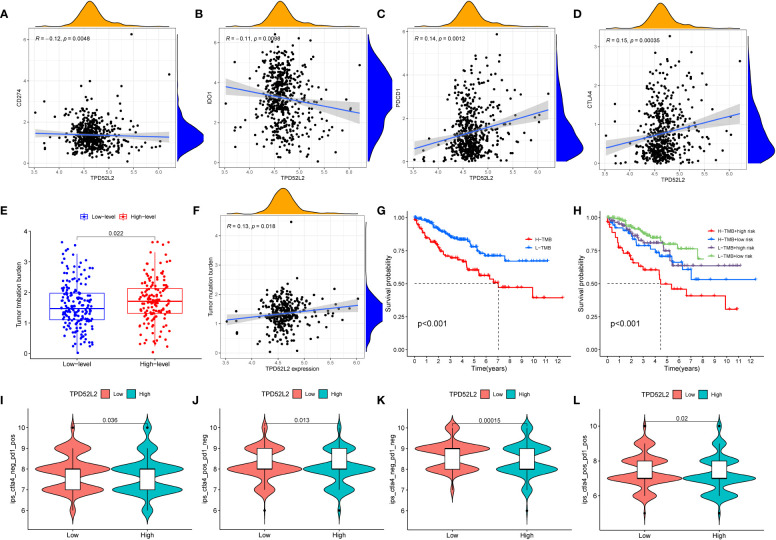
The relationship between TPD52L2 expression and immunotherapy markers. **(A–D)** The correlation between TPD52L2 and common immune checkpoints; **(E)** The difference of TMB between high and low expression TPD52Lgroups; **(F)** Correlation of TPD52L2 expression with TMB; **(G, H)** The relationship between TMB and overall survival of ccRCC patients; **(I–L)** Relationship between TPD52L2 expression and IPS score.

Despite the breakthroughs in immunotherapy for malignancies, only a fraction of patients can respond and benefit from it. Therefore, benefit assessment prior to immunotherapy is necessary. The expression level of immune checkpoints, such as PDL1, is one of the important biomarkers of immunotherapeutic response (23). Therefore, we explored the relationship between the expression of TPD52L2 and immune checkpoints. The results revealed that 17 immune checkpoints were significantly different in both high and low TPD52L2 expression groups. Among them, LGALS9, LAIR1, TNFRSF25, LAG3, TNFRSF8, CD44, TNFRSF4, TNFSF14, TMIGD2, CTLA4, CD276, TNFRSF18 were expressed at higher levels in the TPD52L2 high expression group compared to the low expression group, while TNFSF15, HAVCR2, HHLA2, CD274, and NRP1 were expressed at lower levels, as shown in **Figure S3**. Further, correlation analysis was done for four common immune checkpoints, and the results showed that TPD52L2 expression was negatively correlated with CD274 and IDO1, and positively correlated with PDCD1 and CTLA4, as shown in **Figures 10A–D**.

Tumor mutational load (TMB) is also an important biomarker that is closely associated with prognosis and immunotherapy response in tumor patients (24). Therefore, we explored the relationship between the expression of TPD52L2 and TMB. The results showed that TMB levels were higher in the TPD52L2 high expression group, and TMB was positively correlated with TPD52L2 expression levels, as shown in **Figures 10E, F**. Subsequently, the effect of TMB on the survival of ccRCC patients was compared, and the results showed that patients with high expression of TMB and high expression of TPD52L2 had the worst prognosis, and the effect of high or low TPD52L2 expression on OS of patients was not affected by the status of high or low TMB (**Figures 10G, H**). The above results indicated that the expression of TPD52L2 was closely related to the expression of TMB, CTLA-4 and PD1. To further evaluate the predictive value of TPD52L2 expression on ICIs treatment response. We obtained IPS scores for anti-CTLA-4 and anti-PD-I immunotherapy responses in ccRCC patients through the TCIA platform for comparison. The results showed that the low TPD52L2 group had higher IPS scores when anti-PD1 therapy was used alone, and the IPS scores were higher in the TPD52L2-high expression group when anti-CTLA4 was used alone or both (**Figures 10I–L**).

These studies above indicate that TPD52L2 may not only participate in the immune escape of ccRCC cells, but also may become a potential marker for predicting the efficacy of immunotherapy.

### 3.10 Aberrant expression of TPD52L2 affects targeted therapy for ccRCC

**Figure 11:**
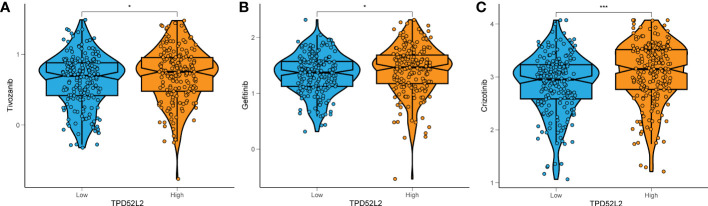
The relationship between TPD52L2 expression level and ccRCC antitumor drug sensitivity. TPD52L2 low expression is more sensitive to **(A)** Tivozanib, **(B)** Gefitinib, **(C)** Crizotinib. *P<0.05, ***P<0.001.

Currently, Tyrosine kinase inhibitors (TKIs) are the first-line agents for the treatment of advanced ccRCC (25). To this end, we collected data on TKIs-targeted drugs in ccRCC treatment through the NCI-60 cancer cell line database and compared the expression of TPD52L2 with drug sensitivity. The results showed that the half maximal inhibitory concentration (IC50) of Tivozanib, Gefitinib, and Crizotinib was lower in the TPD52L2 low expression group as shown in **Figures 11A–C**. This suggests that TPD52L2 may be a potential biomarker for the efficacy of TKIs in ccRCC patients. In general, these findings provide some guidance and evidence for the rational medication of ccRCC patients.

### 3.11 TPD52L2 promotes the proliferation, migration, and invasion of ccRCC cell

**Figure 12:**
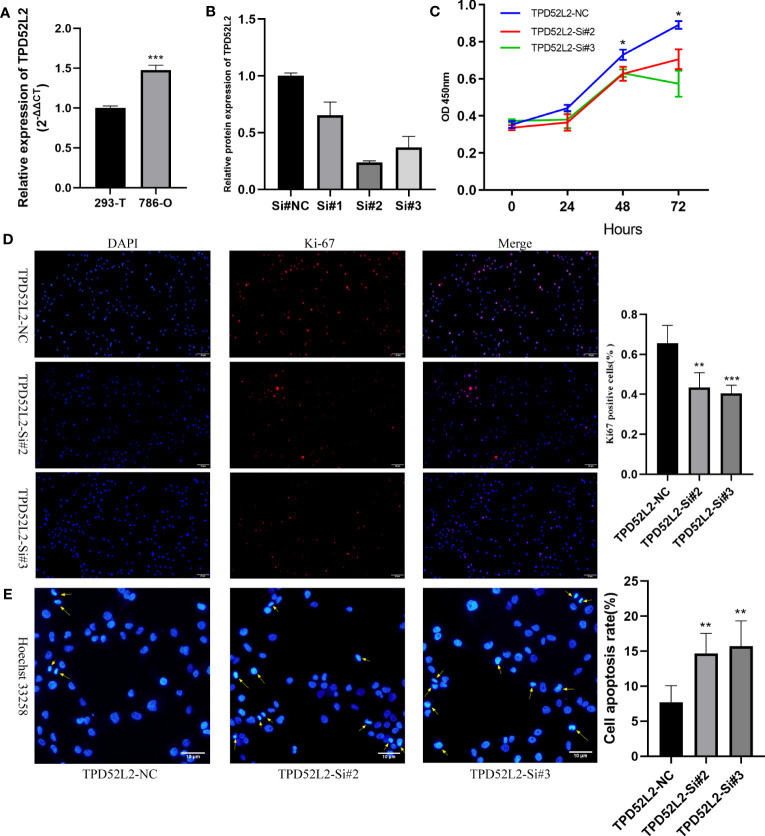
Effect of TPD52L2 on proliferation and apoptosis of 786-O cells. **(A)** RT-qPCR to clarify the expression level of TPD52L2 in 786-O; **(B)** RT-qPCR to detect si-RNA transfection efficiency; **(C)** CCK8 assay to detect the change on proliferation ability after knockdown of TPD52L2; **(D)** Immunofluorescence to detect the difference of KI-67 expression; **(E)** Hoechst 33258 detects changes in apoptosis. *P<0.05, **P<0.01, ***P<0.001.

**Figure 13:**
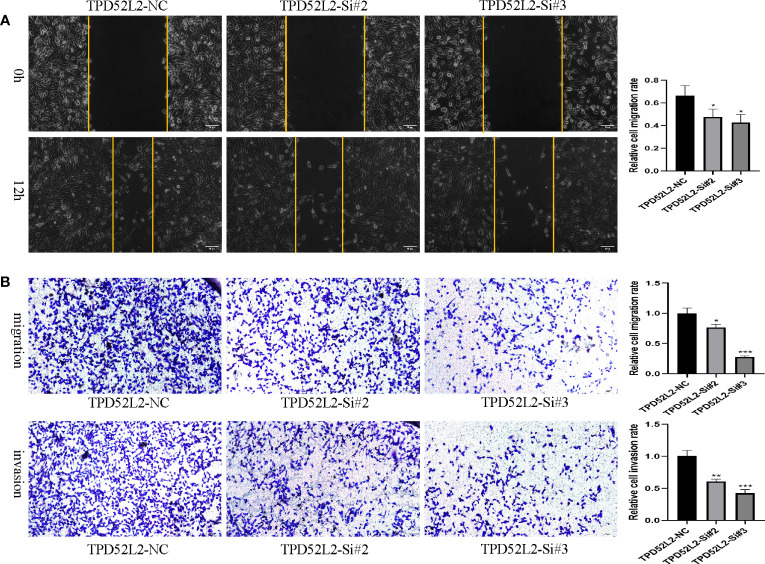
Effect of TPD52L2 on migration and invasion of 786-O cells. **(A)** Wound healing assay to detect the effect of knockdown of TPD52L2 on the migration of 786-O cells; **(B)** Transwell assay to detect the effect of knockdown TPD52L2 on the migration and invasion of 786-O cells. *P<0.05, **P<0.01, ***P<0.001.

To determine whether TPD52L2 affects the malignant biological behavior of ccRCC cell lines, we first confirmed the relative upregulation of TPD52L2 expression in the renal clear cell carcinoma cell line 786-O using RT-qPCR (**Figure 12A**). Next, we used siRNA knockdown to decrease TPD52L2 expression levels in 786-O cells (**Figure 12B**) and assessed changes in cell proliferation, apoptosis, migration, and invasion. Ultimately, the CCK8 experiment results showed that knockdown of TPD52L2 significantly inhibited cell proliferation (**Figure 12C**). Consistently, the proportion of Ki-67-positive cells, a proliferation marker, decreased after knockdown of TPD52L2 (**Figure 12D**). Additionally, Hoechst 33258 staining showed that the rate of cell apoptosis increased after TPD52L2 knockdown (**Figure 12E**). Furthermore, we conducted scratch and Transwell experiments, which demonstrated that the migration and invasion abilities of 786-O cells were significantly reduced after TPD52L2 knockdown (**Figures 13A, B**). Overall, the results suggest that the abnormally high expression of TPD52L2 can promote the malignant phenotype of renal clear cell carcinoma cells, which further validates our bioinformatics analysis results.

## 4 Discussion

In recent years, with clinical practice and prognostic follow-up, it seems that TMN staging alone can no longer fully satisfy the diagnosis and treatment of tumor patients (26). Therefore, more and more oncology guidelines have incorporated multiple novel biomarkers into the prognostic evaluation system of patients, such as breast cancer, prostate cancer, colorectal cancer (27–30). Unfortunately, however, the current diagnosis and prognosis evaluation of ccRCC still lacks effective molecular markers. Therefore, continued efforts to explore novel molecular markers of ccRCC are of great significance.

Obviously, biomarkers require a systematic and rigorous process from initial discovery to final application to the clinic. And most tumor biomarkers, when applied to the clinic, rely on their specific differential levels (31). Therefore, exploring DEGs and exploring their relationship with clinical features and patient prognosis are key fundamental steps in the development of biomarkers. In the present study, we first clarified that TPD52L2 expression was abnormally elevated in ccRCC tissues, either by mRNA or protein. And the results were also confirmed by RT qPCR and proteomic sequencing data. This suggests that TPD52L2 may play an important regulatory role in the pathological process of ccRCC, as abnormal gene expression is one of the important drivers of tumorigenesis and progression (32, 33). Subsequently, clinical correlation analysis revealed that the expression level of TPD52L2 increased along with TNM stage and tumor grade. This implies that TPD52L2 may be a pathogenic gene for ccRCC, as it is generally accepted that TMN stage and grade are negatively correlated with the prognosis of tumor patients (34). Subsequent survival and ROC analyses revealed patients with ccRCC who exhibited high expression levels of TPD52L2 had unfavorable OS and PFS. While the AUC of TPD52L2 demonstrated some diagnostic efficacy in predicting OS, its diagnostic value for PFS was relatively low. It is worth noting AUC should not be solely relied upon as the criterion for diagnostic clinical outcome modeling, as a value of 0.65 has been deemed sufficient in certain predictive models (35). For instance, the prostate cancer biomarker PSA exhibits an AUC of approximately 0.66 (36, 37). To enhance the credibility of TPD52L2’s prognostic value, we conducted univariate, multivariate, and Meta-analysis analyses. The findings were promising, indicating TPD52L2 has the potential to function as an autonomous prognostic risk determinant for patients. The results were encouraging and suggested TPD52L2 may serve as an independent prognostic risk factor for ccRCC patients. But given that the prognosis of ccRCC is influenced by several factors, this study additionally integrated TPD52L2 and clinical data to construct nomogram. Encouragingly, the ROC curves demonstrated the nomogram exhibited good diagnostic efficacy in predicting OS. Meanwhile, some scholars have also reported that TPD52L2 is involved in the pathological process of other tumor as a pathogenic gene (38). Consequently, based on the above discussion, we have reason to believe that TPD52L2 is a pathogenic gene in the pathological process of ccRCC and a biomarker of unfavorable prognosis of patients.

A gene’s function is often achieved through the cooperation of genes with which it is co expressed and interacting (39). Co-expression analysis revealed that TPD52L2 was negatively correlated with VWA8, OSBPL1A, ACADSB, and PURA, and positively correlated with SLC35C2, DNTTIP1, SLC52A2, ARFGAP1, and MRGBP. Notably, ACADSB, OSBPL1A, and PURA are downregulated in a variety of tumors, including ccRCC, leading to disease progression (40–42), while DNTTIP1, SLC52A2, and MRGBP are upregulated in various tumors and act as pathogenic genes (43–45). It is closely related to the expression pattern of some oncogenes, which further indicates that TPD52L2 may play an oncogene role in the pathological process of ccRCC. Exploring the regulatory mechanism of TPD52L2 in the pathological process of ccRCC will help to further expand the understanding of the pathological process of ccRCC. Therefore, signaling pathways potentially regulated by TPD52L2 were explored through gene enrichment analysis. The results showed that TPD52L2 might regulate multiple tumor progression-related signaling pathways, such as the PI3K−Akt signaling pathway, IL−17 signaling pathway, Wnt signaling pathway, and Hippo signaling pathway. And these pathways are widely recognized for their significant involvement in the regulation of malignant biological behavior in ccRCC cellst (46–48). Notably, these pathways are also involved in the formation of the immune microenvironment of the tumor. For example, PI3K/AKT signaling pathway, Wnt signaling pathway, Hippo signaling pathway, and IL-17 signaling pathway all play an important regulatory role in remodeling the immune microenvironment, reprogramming of T cells and macrophages, and reprogramming of tumor-associated fibroblasts (49–52). In general, TPD52L2 may participate in the pathological evolution of ccRCC by regulating the malignant biological behavior and immune activity of tumor cells.

Given that TPD52L2 is involved in the regulation of several immune-related signaling pathways. We further explored its relationship with the immune microenvironment of ccRCC. As an important component of the immune microenvironment, the composition and abundance of immune cells strongly influence tumor progression and the effectiveness of immunotherapy (53). The current study found that ccRCC patients with high expression of TPD52L2 had a higher abundance of immune cell infiltration in the TME. It is worth noting that immune cells are also involved in tumor progression and affect patient prognosis. For example, Treg cells are involved in the immune escape of tumor cells and are associated with poor prognosis in kidney and cervical cancers (54). M1 macrophages can induce anti-tumor immune responses and ultimately inhibit tumor growth (55). Consistent with this, our results showed that the expression level of TPD52L2 in ccRCC TME was directly proportional to the abundance of Treg infiltration and inversely proportional to the abundance of M1 macrophage infiltration. In summary, it can be considered that TPD52L2 may be involved in disease progression by participating in the formation of complex immune microcircles in ccRCC.

The prognosis of cancer patients has been significantly enhanced by anti-tumor immunotherapy. However, several challenges persist, including immune toxicity, tumor heterogeneity, drug resistance, and non-responsiveness. Consequently, the identification of personalized immunotherapy biomarkers is of utmost importance. Currently, TMB and PD-L1 serve as reliable biomarkers, with elevated levels correlating with improved responses to immune therapy (23, 56). Current study found TPD52L2 expression was positively correlated with TMB and negatively correlated with PD-L1. And in the clinical cohort analysis, higher TPD52L2 expression was observed in patients who responded to PD1 inhibitors. Overall, ccRCC patients with high TPD52L2 expression may have a better response to immunotherapy. However, the negative correlation between TPD52L2 and PD-L1 expression challenges this finding. Nevertheless, evidence suggests positive anti-tumor responses to immunotherapy can still occur in patients with low or absent PD1/PDL1 expression (57). Of course, this also indicates the task of identifying effective biomarkers for cancer immunotherapy is formidable and far-reaching.

Despite the comprehensive analysis and *in vitro* experiments conducted in this study, which revealed TPD52L2 as an unfavorable prognostic biomarker for ccRCC and its promotion of malignant biological behavior in ccRCC cells, its practical application in clinical remains a challenge. Consequently, future endeavors should focus on the following areas: Firstly, gathering extensive clinical tissue samples from ccRCC patients across multiple centers, including blood, urine, cancerous, and adjacent non-cancerous samples, to assess the expression level of TPD52L2. Secondly, acquiring clinical and prognostic data from a large and diverse patient population across multiple centers to enhance the accuracy and dependability of prognostic evaluations. Besides, conducting prospective studies to assess the clinical utility of TPD52L2 is also recommended.

## 5 Conclusion

In general, this study found for the first time that the expression of TPD52L2 in ccRCC was increased. As a pathogenic gene involved in the malignant pathological process of ccRCC, it was an independent diagnostic risk factor for the prognosis of patients. In addition, TPD52L2 may participate in the pathological process of ccRCC by participating in the formation of an inhibitory immune microenvironment. More interestingly, we also found that TPD52L2 can also be used as a biomarker, which may have a synergistic effect with existing immunotherapy markers such as TMB to help patients with personalized diagnosis and treatment.

## Data availability statement

The datasets presented in this study can be found in online repositories. The names of the repository/repositories and accession number(s) can be found in the article/**Supplementary Material**.

## Ethics statement

All patients signed informed consent. This study was approved by the Ethics Committee of Lanzhou University Second Hospital (No.2017A-054).

## Author contributions

HW was responsible for compiling all experimental results and writing the first draft. ZL, YG and PS conceived and designed the project. YD is responsible for analyzing the data. XC and SG are responsible for collecting and downloading data. All authors contributed to the article and approved the submitted version.

## Supplementary material

Supplementary Figure 1 Expression of TPD52L2 in relation to TMB in a variety of tumors.

Supplementary Figure 2 Correlation of TPD52L2 with immune microenvironment scores and immune cells. **(A–C)** Correlation of TPD52L2 expression with, immune cells, stromal cells, and tumor purity; **(B–G)** Correlation of TPD52L2 expression with immune cells.

Supplementary Figure 3 Differential expression of multiple immune checkpoints in high and low TPD52L2 expression groups.
