# Supplementary figures and images for: TPD52L2 as a potential prognostic and immunotherapy biomarker in clear cell renal cell carcinoma
Source: Front Oncol. 2023 Nov 23;13:1210910. doi: 10.3389/fonc.2023.1210910 (PMC10701739; doi:10.3389/fonc.2023.1210910)

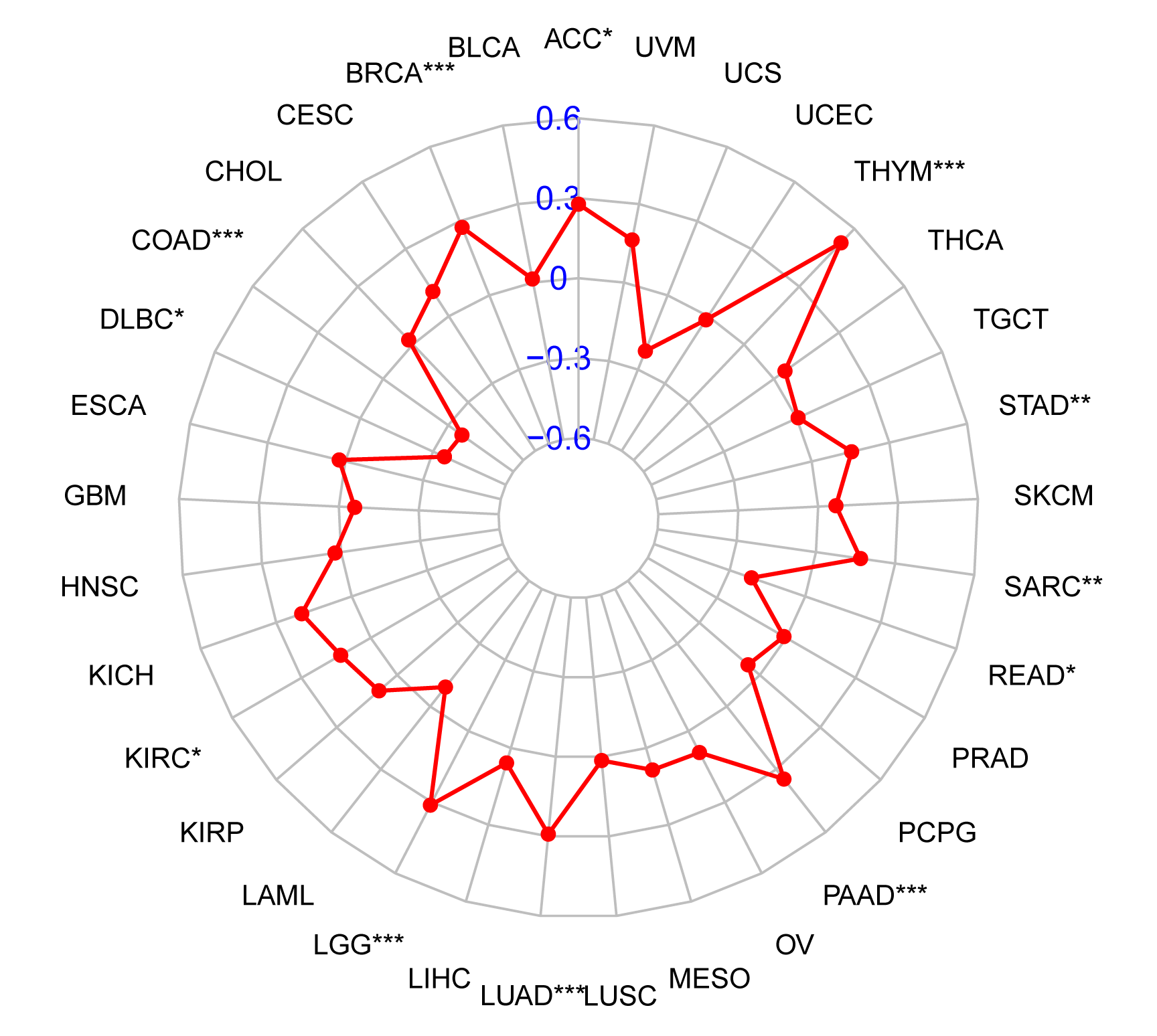

Supplement: Supplementary Figure 1 — Expression of TPD52L2 in relation to TMB in a variety of tumors. [file Image_1.tif]

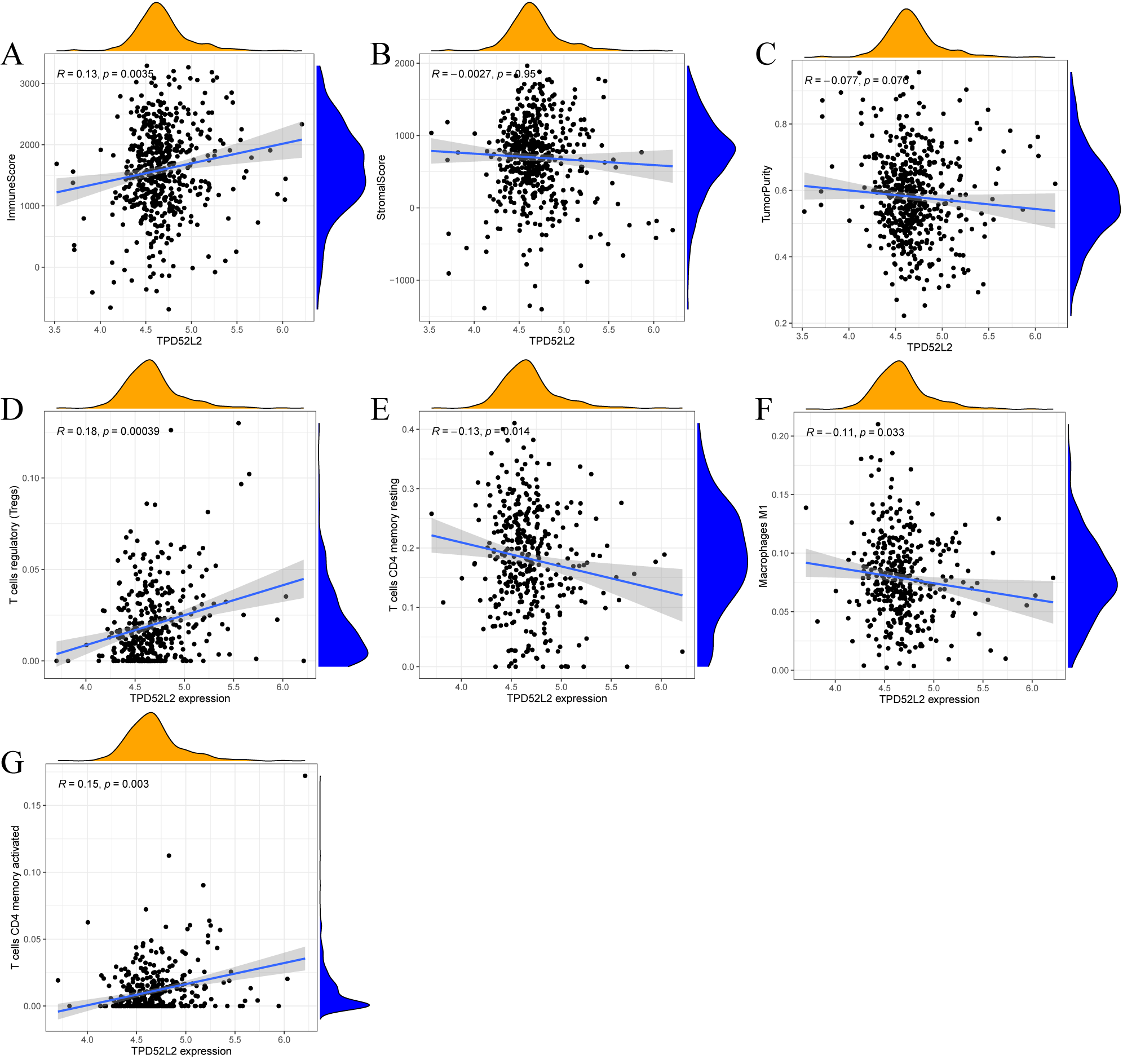

Supplement: Supplementary Figure 2 — Correlation of TPD52L2 with immune microenvironment scores and immune cells. (A–C) Correlation of TPD52L2 expression with, immune cells, stromal cells, and tumor purity; (B–G) Correlation of TPD52L2 expression with immune cells. [file Image_2.tif]

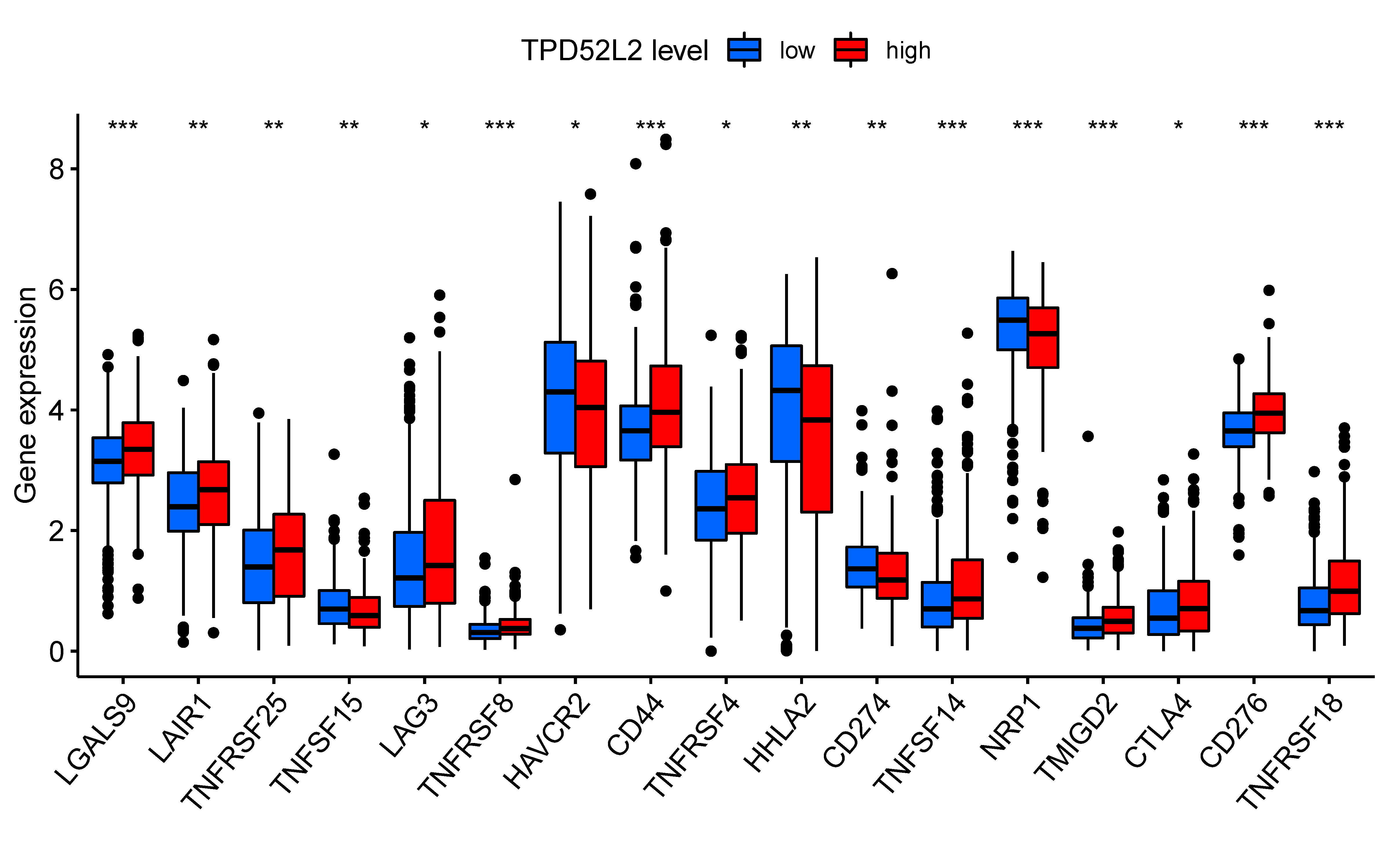

Supplement: Supplementary Figure 3 — Differential expression of multiple immune checkpoints in high and low TPD52L2 expression groups. [file Image_3.tif]
