# Supplementary material for: TPD52L2 as a potential prognostic and immunotherapy biomarker in clear cell renal cell carcinoma
Source: Front Oncol. 2023 Nov 23;13:1210910. doi: 10.3389/fonc.2023.1210910 (PMC10701739; doi:10.3389/fonc.2023.1210910)
Supplement: Supplementary file 4 [file Table_1.docx]

**Table S1 The detailed clinical features of KIRC patients in TCGA**

| **Covariates** | **Type** | **Total** | **Percentages (%)** |
| --- | --- | --- | --- |
| Age | <=65 | 352 | 65.55% |
|  | >65 | 185 | 34.45% |
| Gender | Female | 191 | 35.57% |
|  | Male | 346 | 64.43% |
| Grade | G1 | 14 | 2.61% |
|  | G2 | 230 | 42.83% |
|  | G3 | 207 | 38.55% |
|  | G4 | 78 | 14.53% |
|  | unknow | 8 | 1.49% |
| Stage | Stage I | 269 | 50.09% |
|  | Stage II | 57 | 10.61% |
|  | Stage III | 125 | 23.28% |
|  | Stage IV | 83 | 15.46% |
|  | unknow | 3 | 0.56% |
| T | T1 | 275 | 51.21% |
|  | T2 | 69 | 12.85% |
|  | T3 | 182 | 33.89% |
|  | T4 | 11 | 2.05% |
| Metastasis | M0 | 79 | 14.71% |
|  | M1 | 426 | 79.33% |
|  | unknow | 32 | 5.96% |
| N | N0 | 240 | 44.69% |
|  | N1 | 17 | 3.17% |
|  | unknow | 280 | 52.14% |

**Table S2 The detailed clinical features of KIRC patients with DNA methylation**

| **Covariates** | **Type** | **Total** | **Percentages (%)** |
| --- | --- | --- | --- |
| Age | <=65 | 207 | 65.3% |
|  | >65 | 110 | 34.7% |
| Gender | Female | 112 | 35.33% |
|  | Male | 205 | 64.67% |
| Grade | G1 | 9 | 2.84% |
|  | G2 | 133 | 41.96% |
|  | G3 | 123 | 38.8% |
|  | G4 | 48 | 15.14% |
|  | unknow | 4 | 1.26% |
| Stage | Stage I | 155 | 48.9% |
|  | Stage II | 31 | 9.78% |
|  | Stage III | 72 | 22.71% |
|  | Stage IV | 57 | 17.98% |
|  | unknow | 2 | 0.63% |
| T | T1 | 159 | 50.16% |
|  | T2 | 41 | 12.93% |
|  | T3 | 109 | 34.38% |
|  | T4 | 8 | 2.52% |
| M | M0 | 233 | 73.5% |
|  | M1 | 52 | 16.4% |
|  | unknow | 32 | 10.09% |
| N | N0 | 133 | 41.96% |
|  | N1 | 8 | 2.52% |
|  | unknow | 176 | 55.52% |

| Gene | sequence (5′–3′) |
| --- | --- |
| siRNA-NC (sense) | UUCUCCGAACGUGUCACGUTT |
| siRNA-NC (antisense) | ACGUGACACGUUCGGAGAATT |
| siRNA-1 (sense) | GCCAAGAUAUCAACCUGAATT |
| siRNA-1 (antisense) | UUCAGGUUGAUAUCUUGGCTT |
| siRNA-2 (sense) | GUGACUUCAAGUGUCGGAUTT |
| siRNA-2 (antisense) | AUCCGACACUUGAAGUCACTT |
| siRNA-3 (sense) | GCAUGAGUAUCUGGGAUGUTT |
| siRNA-3 (antisense) | ACAUCCCAGAUACUCAUGCTT |

**Table S3 The specific sequences of siRNA**
